# The effect of the COVID-19 pandemic on the prescribing of opioid and opioid use disorder medications within an academic medical center in California

**DOI:** 10.3389/fpubh.2023.1105681

**Published:** 2023-06-07

**Authors:** Armen K. Fstkchian, Jessa Koch, Khaled Bahjri, Lisa T. Hong

**Affiliations:** ^1^Adventist Health White Memorial, Los Angeles, CA, United States; ^2^Loma Linda University School of Pharmacy, Loma Linda, CA, United States

**Keywords:** opioid, opioid-use disorder, pandemic, morphine-milligram equivalents, COVID-19

## Abstract

**Introduction:**

The COVID-19 pandemic impacted healthcare operations affecting many patients with chronic pain and substance use disorder. Our study aimed to evaluate the effects of the COVID-19 pandemic on opioid and opioid use disorder (OUD) medication prescribing practices within a large academic health system in southern California.

**Methods:**

This retrospective cohort study included patients who received a prescription for chronic opioids or therapy for OUD between November 1, 2019 and September 1, 2020. The date range was divided into five specific time periods during the pandemic: November through December 2019 (pre-COVID and reference period), January through February 2020 (early COVID), March through April 2020 (policy/guidance change period), May through June 2020 (early post-guidance period), and July through August 2020 (late post-guidance period). The primary outcome was change in morphine milligram equivalents (MME) prescribed. Secondary outcomes included encounter type, mode of prescription ordering, naloxone prescriptions, and urine drug screen obtainment.

**Results:**

The cohort included 100 patients divided among the designated time periods. Seventy-percent of patients received opioids for chronic non-malignant pain and 10% received therapy for OUD. Although there were numerical increases in MMEs prescribed, no significant changes were seen in the MMEs prescribed at any timepoint relative to the pre-COVID timeframe despite reduced in-person visits, increased video and telephone encounters and increased electronic prescription utilization. Subgroup analyses of those with chronic pain only or OUD had similar findings.

**Conclusion:**

It appears that, generally, prescribing practices were sustained despite the various phases of the pandemic including transitions to and from telemedicine.

## Introduction

As of August 2022, Coronavirus Disease 2019 (COVID-19) has led to over 90 million cases in the United States (US) and over 1-million deaths (1). The pandemic has affected many aspects of our nation’s infrastructure including the labor force, mental health, and healthcare (2–5). Healthcare institutions have experienced significant operational changes as states and local governments adjust a range of policies to reduce COVID-19 transmission (6). These measures have included canceling or postponing elective procedures, limiting outpatient visits, converting to telemedicine for non-urgent health services, and enacting exemptions for prescribing and refilling controlled substances (7–10). The National Bureau of Economic Research found a 40% decline in outpatient visits from the first week of March to early April 2020 (11). Delays or cancelations of healthcare visits may have deleterious effects on patient health outcomes. Patients living with chronic pain and OUD are particularly susceptible to adverse outcomes as their care is dependent upon access to in-person services, such as physical therapy, interventional procedures, and initiation of specific medications (12–15).

While prescriptions for controlled substances issued electronically must generally be predicated on an in-person medical evaluation, the federal Controlled Substances Act contains exceptions during a public health emergency. Thus, on January 31, 2020, when the Secretary of Health and Human Services (HHS) declared COVID-19 a nationwide public health emergency (16), prescribing controlled substances via telemedicine without an initial in-person visit was permitted. Additionally, the Centers for Medicare and Medicaid Services (CMS) expanded telehealth services, allowing providers to be reimbursed at the same rate as an in-person visit (17). In March of 2020, the Substance Abuse and Mental Health Services Administration (SAMHSA) and the Drug Enforcement Agency (DEA) released guidance documents to help providers navigate prescribing controlled substances, including OUD medications, amid the pandemic (18). Several governing bodies provided guidance to managing patients with chronic pain and OUD during the pandemic (18–23). Soon after, on April 16, 2020, CMS issued guidelines to “Opening Up America Again,” allowing for state and local governments to resume in-person nonemergent and non-COVID care if certain criteria were met (24). Specific to California, beginning in May 2020, the governor signed several executive orders, one of which informed local health jurisdictions that they may gradually reopen with modifications (25). Considering the many direct and indirect ways healthcare administration was impacted by the pandemic, the objective of this study was to evaluate the effects of the COVID-19 pandemic on opioid and OUD medication prescribing practices. Previously published studies found significant decreases in the prescribing of these medications among opioid naïve or new patients, who appear to be vulnerable to reduced access to care (5, 26). We aimed to determine how such prescribing is affected within a large academic health system amid a public health emergency among individuals with chronic opioid or OUD prescriptions.

## Methods

### Procedures

**Figure 1:**
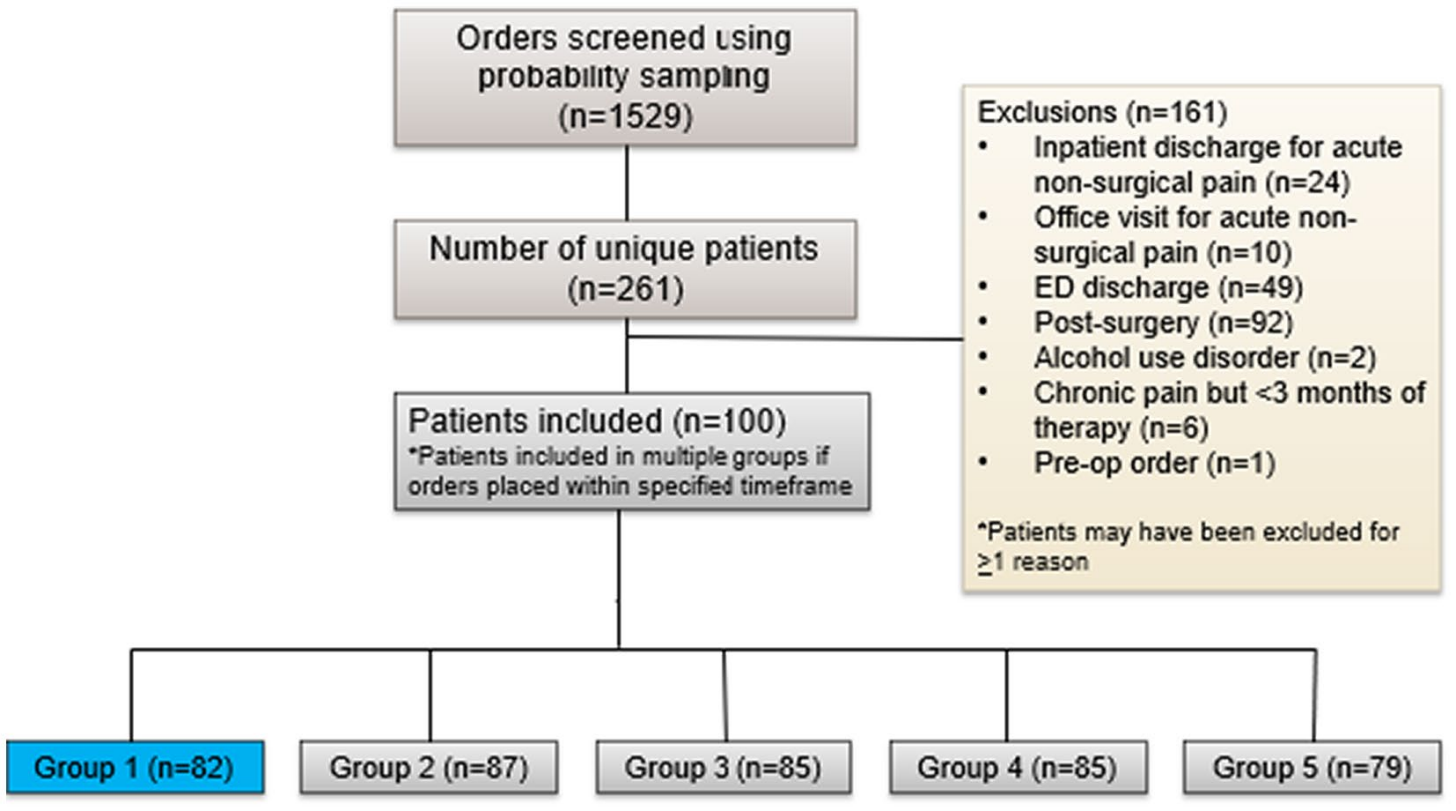
The number of patients screened for inclusion in the study and reasons for exclusion leading to the final study population.

This study was approved by the local Institutional Review Board and was a retrospective cohort study that included patients at least 18 years old who received an outpatient prescription for an opioid or OUD medication between November 1, 2019, and September 1, 2020. The study institution provides an average of 1 million outpatient visits per year. We excluded patients on opioid or OUD therapy for fewer than 3 months and those prescribed opioids for post-surgical pain or after an emergency department discharge. Data inquiry was made for all outpatient opioid prescription orders including fentanyl, hydrocodone, hydromorphone, morphine, oxycodone, oxymorphone, and tapentadol as well as OUD medications: buprenorphine (with or without naloxone) and methadone. A random number generator was used for probability sampling to screen orders for study inclusion (Figure 1). Most orders were excluded as they were short-term opioid prescriptions prescribed after a surgical procedure. Additionally, identical orders for the same patient within the same time frame were nullified and use of the random number generator was repeated for the next selection.

Included patients were distributed among five different timeframes, depending on which timeframe they received an opioid prescription. The different time periods represent the different phases of the pandemic. Group 1 represents individuals who received a prescription(s) in November or December 2019, which is designated as the pre-COVID time period before the public health emergency was announced and serves as the reference group to which all other groups are compared. Group 2 represents patients who received a prescription(s) in January or February 2020 and is designated as the early COVID timeframe when the public health emergency was announced but policies and guidance documents were not implemented or released. Group 3 represents patients who received a prescription(s) in March or April 2020 and is designated as the policy change period when stay-at-home orders were enacted, outpatient services were suspended, and policies regarding prescribing practices were implemented. Group 4 are patients who received a prescription(s) May or June 2020 and is designated as the early post-guidance period as it represents the time period after aforementioned policies were implemented and when guidelines were released regarding reopening of outpatient and non-urgent medical services. Lastly, Group 5 are patients who received a prescription(s) in July or August 2020 and is designated as the late post-guidance period representing months after policy changes occurred and outpatient services began reopening. Of note, if individuals had medication orders across the time frames, they were included in each group for which they had an order.

The objective of this study was to evaluate the effects of the COVID-19 pandemic on opioid and OUD medication prescribing practices within an academic medical center in southern California. The primary outcome was change in morphine milligram equivalents (MME) defined as the difference between daily MME prescribed to the patient before the respective timeframe and the daily MME of the last prescription ordered in that same timeframe. Secondary outcomes included encounter type (in-person, video, telephone, or refill request encounters, where a prescription was predicated on a message or phone call from the patient requesting a refill), mode of prescription ordering (electronic, print, or both), naloxone prescriptions, and urine drug screen (UDS) obtainment. Outcomes of groups 2–5 were compared to the reference group; group 1.

### Statistical analysis

For descriptive statistics, categorical variables were reported as numbers and percentages while continuous variables were reported as mean ± SD or median with range, depending on the presence of outliers in the data. The mean change in MMEs between the groups was compared with a one-way ANOVA test. Secondary outcomes were compared with a Chi-square test except when comparing medians, for which a Kruskal-Wallis test was utilized. Sub-group analyses were conducted for the primary outcome among individuals with chronic pain only, OUD only, and individuals who received a prescription in group 1 plus groups 2 through 5. For the latter subgroup, a mixed model’s analysis was utilized to follow individuals across different timepoints and compare the mean difference in MME in each group using group 1 as the reference point. Standardized residual plots were used to assess for outliers. Due to multiple testing, these values of *p* are adjusted for the alpha error inflation using Bonferroni corrections. IBM SPSS Statistics for Windows (Version 27.0) was utilized for data-analysis.

## Results

A total of 100 unique patients were included. Most patients (approximately 65%) were female with a mean age of 57 years. The most common indications for opioid prescriptions were chronic non-malignant pain syndromes with over half of the patients reporting severe pain (≥7 out of 10). The majority of clinicians were in primary care clinics (about 60%) and most prescribed short-acting opioids as needed (about 64%). More than half of patients in each group were concomitantly taking non-opioid pain medications including topical analgesics, anti-epileptics, and other medications used to treat neuropathy. Greater than 40% of patients in each group were also taking antidepressants, and about 10% had a concurrent benzodiazepine. Almost all patients (94%) were insured during the time of their encounter, though over one-quarter were unemployed. No significant differences in baseline characteristics were observed in each group relative to group 1 (Table 1).

**Table 1:** Baseline characteristics.

		Groups					*p* value
		1	2	3	4	5	
		*n* = 82	*n* = 87	*n* = 85	*n* = 85	*n* = 79	
Gender	Male	30 (37.0)	31 (36.0)	27 (32.1)	31 (36.9)	27 (34.6)	0.963
	Female	52 (63.0)	56 (64.0)	58 (67.9)	54 (63.1)	52 (65.4)	
Age, mean ± SD		57.5 ± 16.3	56.9 ± 16.2	57.0 ± 17.8	56.8 ± 16.2	57.8 ± 16.6	0.994
Post-visit Rx day supply, median (range)		30 (3–90)	30 (4–90)	30 (5–90)	30 (5–90)	30 (7–90)	0.198
Verbal PRS*	No pain (score 0)	9 (16.4)	8 (13.6)	9 (16.4)	4 (7.5)	5 (10.4)	0.939
	Mild (score 1–3)	4 (7.3)	3 (5.1)	4 (7.3)	3 (5.7)	2 (4.2)	
	Moderate (score 4–6)	14 (25.5)	18 (30.5)	12 (21.8)	12 (22.6)	12 (25.0)	
	Severe (score ≥ 7)	28 (50.9)	30 (50.8)	30 (54.5)	34 (64.2)	29 (60.4)	
Indication	Chronic non-malignant pain	58 (72.5)	58 (68.2)	60 (72.3)	60 (72.3)	58 (75.3)	0.994
	Cancer related pain	15 (18.8)	18 (21.2)	15 (18.1)	14 (16.9)	13 (16.9)	
	Opioid use disorder	7 (8.8)	9 (10.6)	8 (9.6)	9 (10.8)	6 (7.8)	
Control Substance Type	Scheduled	9 (11.1)	12 (14.1)	10 (11.8)	13 (15.3)	13 (16.5)	0.882
	Short-acting PRN	54 (66.7)	58 (68.2)	51 (61.4)	53 (63.1)	48 (61.5)	
	Scheduled + PRN combined	18 (22.0)	15 (17.2)	22 (26.5)	17 (20.2)	16 (20.3)	
Concomitant medications	NSAIDs	17 (22.1)	20 (24.4)	19 (23.5)	20 (25.3)	23 (31.5)	0.718
	Anti-depressants	35 (45.5)	38 (46.3)	40 (49.4)	40 (50.6)	35 (47.9)	0.966
	Tramadol	1 (1.3)	3 (3.7)	2 (2.5)	3 (3.8)	3 (4.1)	0.842
	Muscle relaxants	22 (28.6)	24 (29.3)	22 (27.2)	27 (34.2)	23 (31.5)	0.888
	Non-opioid pain meds	45 (58.4)	20 (35.1)	47 (58.0)	49 (62.0)	41 (57.7)	0.221
	Z-Hypnotics	3 (3.9)	3 (3.7)	4 (4.9)	4 (5.1)	3 (4.2)	0.990
	Benzodiazepines	6 (7.8)	8 (9.8)	9 (11.1)	8 (10.1)	7 (9.6)	0.971
Provider Specialty	Behavioral health	5 (6.1)	5 (5.7)	6 (7.1)	8 (9.4)	5 (6.3)	0.998
	Primary care	50 (61.0)	55 (63.2)	51 (60.0)	54 (63.5)	50 (63.3)	
	Pain/Palliative care/PM&R	20 (24.4)	19 (21.8)	20 (23.5)	16 (18.8)	21 (26.6)	
	Other	6 (7.3)	7 (8.0)	7 (8.2)	6 (7.1)	2 (2.5)	
Co-morbidities	Trauma	6 (7.9)	6 (7.5)	7 (8.6)	6 (7.8)	5 (6.9)	0.997
	Mood disorder	38 (50.0)	41 (50.6)	42 (52.5)	39 (50.0)	38 (52.1)	0.997
	Psychotic disorder	5 (6.5)	8 (9.8)	8 (9.9)	8 (10.1)	7 (9.6)	0.932
	Anxiety disorder	29 (37.7)	31 (37.8)	32 (39.5)	31 (39.2)	29 (39.7)	0.998
	Suicidal ideation	3 (3.9)	4 (4.9)	5 (6.3)	5 (6.4)	6 (8.3)	0.832
	Renal impairment	6 (7.9)	4 (4.9)	5 (6.3)	4 (5.1)	4 (5.5)	0.938
	Liver impairment	3 (3.9)	3 (3.7)	2 (2.5)	2 (2.6)	4 (5.5)	0.863
Employment	Employed	19 (48.7)	19 (48.7)	22 (52.4)	20 (50.0)	19 (52.8)	0.998
	Unemployed	10 (25.6)	12 (30.8)	12 (28.6)	12 (30.0)	9 (25.0)	
	Retired	6 (15.4)	5 (12.8)	6 (14.3)	4 (10.0)	6 (16.7)	
	Disability	4 (10.3)	3 (7.7)	2 (4.8)	4 (10.0)	2 (5.6)	
Health insurance		71 (92.2)	75 (91.5)	76 (95.0)	74 (94.9)	70 (95.9)	0.732

This table provides the comparison of baseline characteristics among patients in each of the five time periods

All values listed as *N* (%) unless otherwise noted. MME, morphine milliequivalents; OUD, opioid use disorder; PM&R, physical medicine and rehabilitation; PRN, as needed; PRS, pain rating scale; Rx, prescription; SD, standard deviation; and TDD, total daily dose. ^*^Not all patients had a pain score documented and percentages reported reflect total number in each group.

**Figure 2:**
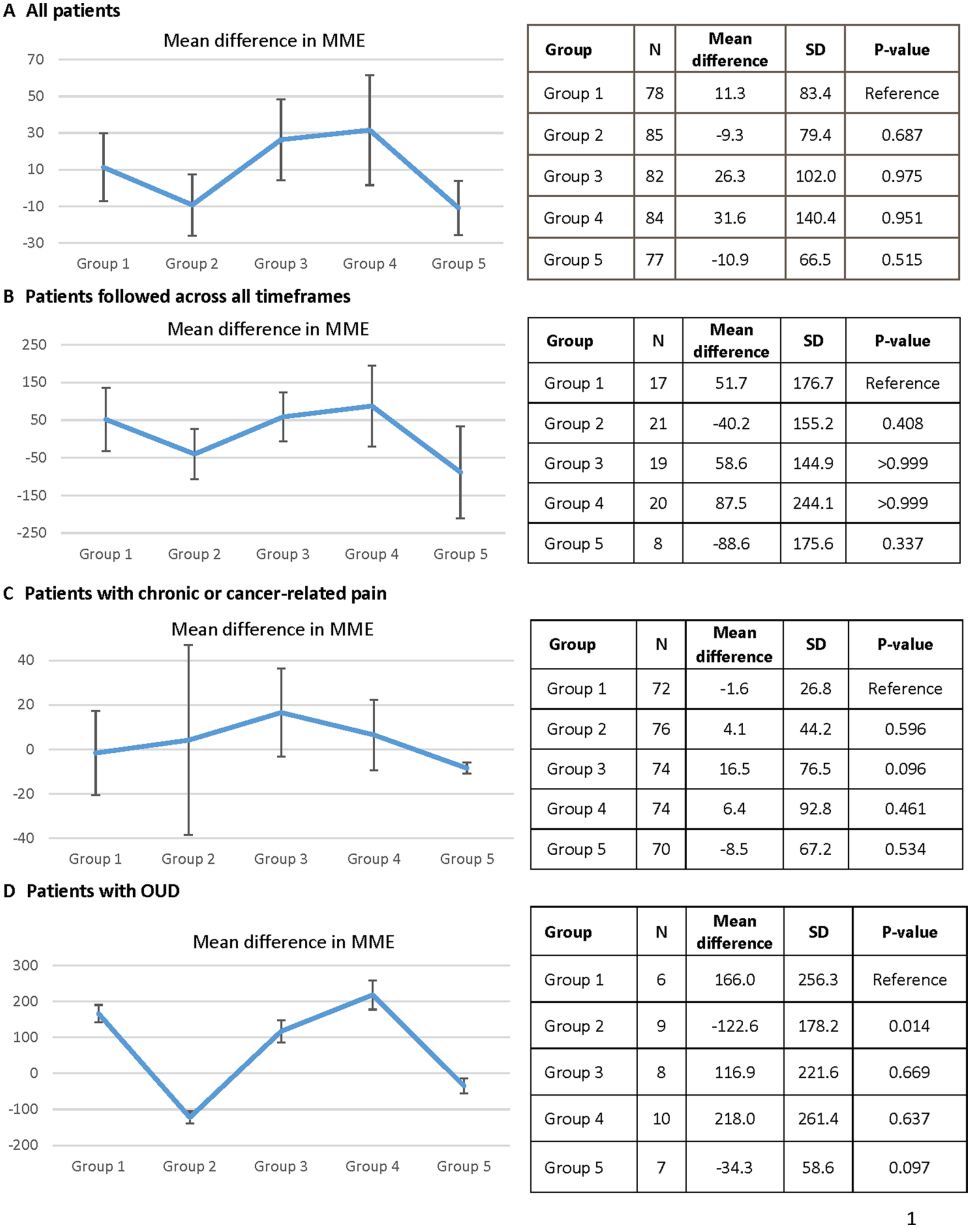
The difference in morphine milligram equivalents measured in each group over the five study periods. Graph **(A)** is an analysis of all patients included in the study. Graph **(B)** is an analysis only of patients who had prescription in each of the five time periods. Graph **(C)** is an analysis of patients prescribed opioids for chronic, non-malignant pain. Graph **(D)** is an analysis of patients only prescribed buprenorphine, naloxone, or methadone for opioid use disorder. MME, morphine milligram equivalents; OUD, opioid use disorder.

For the primary endpoint, a numerical increase in mean daily MMEs was prescribed in groups 1 (11.3 ± 83.4), 3 (26.3 ± 102.0), and 4 (31.6 ± 140.4) and a mean decrease in daily MMEs was prescribed in groups 2 (−9.3 ± 79.4) and 5 (−10.9 ± 66.5), but no significant changes were observed relative to group 1 (Figure 2A). In a subgroup analysis including individuals consistently seen by their provider through the pandemic [patients in group 1 who also had prescription(s) in the other time frames], the pattern of increased MMEs in groups 3 and 4 and a decrease in MMEs in group 5 was consistent with the original analysis (Figure 2B). However, the findings were similarly nonsignificant. Among patients with OUD, a significant decrease in MMEs was observed between groups 1 and 2 (166 ± 256.3 vs. -122.6 ± 178.2; *p* = 0.014) with no other significant differences in prescribed MMEs and similar overall trends to the primary analysis (Figure 2C). When evaluating the 73 (90%) individuals with chronic pain only, an uptrend was seen with MMEs prescribed in group 3 (Figure 2D). In contrast to other analyses, group 2 followed an upward trend in MMEs and group 4 trended downward.

In-person visits significantly decreased and telephone and video encounters increased with the exception of group 2 where there was no difference in the number of video visits, relative to group 1 (both 0%). No difference was seen in refill request encounters nor naloxone prescribing. Providers prescribed controlled substances electronically significantly more as time passed through the pandemic and there was a significant decrease in UDS obtainment at each visit (Table 2).

**Table 2:** Secondary outcomes.

		Groups					*p* value
		1	2	3	4	5	
		*n* = 82	*n* = 87	*n* = 85	*n* = 85	*n* = 79	
Encounter type	In-person visit	62 (76.5)	62 (72.1)	31 (36.9)	12 (14.3)	12 (15.4)	**<0.001**
	Video	0 (0)	0 (0)	24 (28.6)	29 (34.5)	32 (41.0)	**<0.001**
	Telephone	1 (1.2)	2 (2.3)	31 (36.9)	25 (29.8)	19 (24.4)	**<0.001**
	Refill request	41 (50.6)	42 (48.8)	44 (52.4)	43 (51.2)	42 (53.8)	0.977
	In patient discharge	4 (4.9)	6 (7.0)	3 (3.6)	5 (6.0)	3 (3.8)	0.841
	Multiple visit types	25 (31.3)	25 (29.1)	44 (52.4)	29 (34.5)	27 (34.6)	0.150
Mode of prescription	Electronic	49 (60.5)	53 (61.6)	69 (82.1)	73 (86.9)	70 (89.7)	**<0.001**
	Print	41 (50.6)	38 (44.2)	27 (32.1)	17 (20.2)	17 (21.8)	**<0.001**
	Both electronic and print	9 (11.1)	5 (5.8)	13 (15.5)	6 (7.1)	9 (11.5)	0.244
Naloxone prescriptions		45 (57.7)	50 (61.0)	45 (55.6)	51 (63.8)	45 (60.8)	0.853
UDS at visit		19 (25.0)	21 (26.3)	10 (12.5)	10 (12.8)	8 (10.9)	**0.018**

This table captures differences in encounter type, mode of prescribing, naloxone prescriptions, and obtainment of urine drug screens among the five study periods. UDS, urine drug screen. Bolding is this context is purely for formatting and readability given 4 different charts and tables within one figure.

* All values reported as *n* (%), unless otherwise specified.

## Discussion

No statistically significant changes were observed in MMEs prescribed during various periods of the pandemic. Our results are similar to a recent study that evaluated the effect of the COVID pandemic on the prescribing of opioid analgesics and buprenorphine for OUD and found no changes in total MMEs or units of buprenorphine prescribed among existing patients, defined as those who received a prescription for said medications in the past 365 days (26). We also evaluated changes in MME prescribing per individual rather than total MMEs across the population reflecting a more patient-centered approach than what was previously published.

A numerical decrease was observed in mean MMEs prescribed in the early stages of the pandemic and during the late post-guidance period. Decreases observed among this chronic pain population could possibly be attributable to improved pain control and attempts to taper opioids early in the pandemic. Studies evaluating pharmacy claims databases found significant reductions in the number of opioid prescriptions and individuals filling opioid prescriptions during the pandemic (27, 28). These decreases were likely due to a reduction in elective procedures and non-emergent outpatient visits, thus reducing short-term opioid prescriptions, which were excluded from our analysis. Of note, there were also differences regarding the timeframes as we investigated 2-month windows of time whereas these other studies analyzed trends over a longer duration. Our approach considered specific events during the pandemic that may have influenced prescribing trends. In the late post-guidance period, modifications to stay at home orders and the reopening of different healthcare services increased availability of previously suspended non-emergent outpatient services. Access to routine visits, physical therapy, and social interactions may have improved pain control and treatment of comorbidities (including mental health conditions), reducing the need for opioid therapy. This possibly explains why the mean MMEs trended back down to pre-pandemic levels.

The numerical increases in MME observed during the months of March–June 2020 relative to the months of November–December 2019 may be attributable to the negative effects of the pandemic on mental health conditions, as mental health is intertwined with chronic pain and substance use (29, 30). Stay-at-home orders and the lack of outpatient services, such as interventional and physical therapies, may have also contributed to loss of pain control and the observed increases in prescribed MMEs as these nonpharmacologic treatment modalities play an important role in pain management (23). This potential explanation has been supported by patient-reported reasons for pain regimen changes during the pandemic that included worsening pain, reduced provider access, and increased medication use due to cancelation of physical/psychological therapy (31). Others have similarly demonstrated negative effects of social factors on pain perception and specifically that changes to coping mechanisms for pain are associated with increased medication use (32). Furthermore, social isolation may have contributed to patient deconditioning, which is also a risk factor for loss of pain control, as the population was limited in their outdoor and gym activities (23).

Additionally, the inclusion of OUD medications (buprenorphine and methadone) in the primary analysis may have led to a more exaggerated effect since these medications have a significantly higher MME relative to other common opioids used for pain management. This is supported by the subgroup analysis that included only patients with buprenorphine or methadone prescribed for OUD where the mean differences in MMEs were much larger in magnitude. Trends observed within this small subgroup of patients with OUD were similar to the original analysis suggesting that the changes in MMEs may have been driven largely by changes in OUD prescribed opioids. The observed numerical increases in MMEs are in line with a previous study, which showed a significant increase in the quantity of buprenorphine prescribed per prescription between the months of March and May 2020 (26).

With the vast majority of our patients having health insurance and encounters with their providers in the midst of the pandemic, the overall lack of significant differences in opioid and OUD medication prescribing was likely the result of continued access to care. These findings may not be true among uninsured patients. Unemployment rates surged during this time period of the pandemic with over 20 million Americans being laid off or furloughed (2). As a result, individuals may have lost employer provided health insurance as well as a means to pay for healthcare services, resulting in a loss of access to care, gaps in care, and ability to obtain opioid or OUD prescriptions. Our study cohort only included individuals who were on chronic opioid or OUD therapy for 3 months or more, thus these individuals may have held strong patient-provider relationships with established and regular follow-up, minimizing provider hesitancy to prescribe opioids. The continuity of care was most likely attributed to the increase in telemedicine, allowing patients to maintain access to care. Lastly, published guidance documents and policy changes regarding controlled substance prescribing, in concordance with actions by local governments and health institutions, likely played an important role in ensuring congruent care to patients with chronic pain and OUD.

Similar to our findings, other health systems have also reported increased volume of virtual visits via telephone, video, or a combination between the months of March and April 2020 (33, 34). Collectively, these data and our results suggest that the pandemic may have served as a catalyst for the acceptance, and expansion of telehealth utilization.

Accordingly, with the reduction of in-person visits, obtainment of urine drug screens (UDS) at each encounter decreased significantly. Of note, governing bodies have recommended UDS testing not be mandatory during a public health emergency (35). Active naloxone prescriptions among the cohort remained unchanged in each group. In contrast, a study using pharmacy claims data found significant reductions in naloxone prescriptions filled (28). However, these data may include new patients prescribed opioids rather than only those with chronic opioid prescriptions for pain.

Our study was limited by a small sample size as evidenced by the large standard deviations observed in our results. Given the small number of patients with OUD, independent evaluation of this population may better elucidate the impact of the pandemic on these patients as well as inclusion of our affiliated federally qualified health center, which offers medication assisted treatment and may have been responsible for more of the prescribing during this time. Documentation in the electronic medical record system may be incomplete or inaccurate as what was prescribed may not reflect what was taken by the patient. Furthermore, worsening of substance use, drug overdoses, mental health, and suicidal ideation occurred in the United States during the pandemic (4, 5). These psychosocial aspects and stressors were not specifically investigated in this study and may have impacted the prescribing practices observed. Further assessment of the association between these potential contributing factors with OUD medication and opioid analgesic prescribing is critical, as those living with chronic pain and/or OUD are susceptible to failure in therapy, worsening of mental health, and an increase in mortality related to drug overdose (4, 5).

## Conclusion

Our study provides real-world insight into the continuity of care in patients with chronic pain and OUD amidst a public health emergency at an academic medical center in southern California. We observed no significant changes in MMEs prescribed at different time points in the pandemic relative to pre-pandemic prescribing indicating successful care for this vulnerable patient population during the pandemic despite changes in the approach to care. To further understand the impact of the pandemic on patients with chronic pain and OUD, future research could focus on individual outcomes such as pain control, opioid-related hospitalizations, and OUD relapses, to provide valuable insights into patient experiences and inform clinical practice. Additionally, given the increased use of telemedicine during the pandemic, it may be beneficial to compare the financial and clinical impact of in-person and telehealth visits among patients with chronic pain and/or OUD.

## Data availability statement

The raw data supporting the conclusions of this article will be made available by the authors, without undue reservation.

## Ethics statement

The studies involving human participants were reviewed and approved by Loma Linda University Investigational Review Board. Written informed consent for participation was not required for this study in accordance with the national legislation and the institutional requirements.

## Author contributions

AF, JK, KB, and LH contributed equally to the conceptualization, methodology, validation, and reviewing and editing. Data curation was completed by AF. JK and LH shared project administration and supervision responsibilities. Formal analysis was performed by AF, KB, and LH, and the writing of the original draft was done by AF, JK and LH. All authors contributed to the article and approved the submitted version.

## Conflict of interest

The authors declare that the research was conducted in the absence of any commercial or financial relationships that could be construed as a potential conflict of interest.

## Publisher’s note

All claims expressed in this article are solely those of the authors and do not necessarily represent those of their affiliated organizations, or those of the publisher, the editors and the reviewers. Any product that may be evaluated in this article, or claim that may be made by its manufacturer, is not guaranteed or endorsed by the publisher.

## References

[1] World Health Organization (2022). United States of America. Available at: https://covid19.who.int/region/amro/country/us

[2] Bureau of Labor Statistics United States of America Department of Labor (2020). News Release: The Employment Situation. Available at: https://www.bls.gov/news.release/pdf/empsit.pdf (Accessed June 2022)

[3] Collins S Gunja M Aboulafia G Czyzewics E Kline C Rapport R. An early look at the potential implications of the COVID-19 pandemic for health insurance coverage. (2020) Commonwealth Fund, New York. Available at: https://www.commonwealthfund.org/publications/issue-briefs/2020/jun/implications-covid-19-pandemic-health-insurance-survey

[4] Czeisler ME Lane RI Petrosky E Wiley JF Christensen A Njai R. Mental health, substance use, and suicidal ideation during the COVID-19 pandemic—United States, June 24-30, 2020. MMWR Morb Mortal Wkly Rep. (2020) 69:1049–57. doi: 10.15585/mmwr.mm6932a1, PMID: 32790653 PMC7440121

[5] Faust JS Du C Mayes KD Li SX Lin Z Barnett ML. Mortality from drug overdoses, homicides, unintentional injuries, motor vehicle crashes, and suicides during the pandemic, March-August 2020. JAMA. (2021) 326:84–6. doi: 10.1001/jama.2021.8012, PMID: 34019096 PMC8140390

[6] Gupta S Nguyen TD Rojas FL. Tracking public and private response to the covid-19 epidemic: Evidence from state and local government actions. (2020) Technical report, National Bureau of Economic Research.

[7] US Department of Health and Human Services. Notification of enforcement discretion for telehealth remote communications during the COVID-19 Nationwide public health emergency. (2020) Available at: https://www.hhs.gov/hipaa/for-professionals/special-topics/emergency-preparedness/notification-enforcement-discretion-telehealth/index.html

[8] California Medical Assocation DEA clarifies that controlled substances can be prescribed via telehealth. (2020) Available at: https://www.cmadocs.org/newsroom/news/view/ArticleId/48775/DEA-clarifies-that-controlled-substances-can-be-prescribed-via-telehealth (Accessed March 23, 2020)

[9] Prevoznik T. U.S. Department of Justice: drug enforcement agency. DEA SAMHSA buprenorphine telemedicine. (2020) Available at: https://www.deadiversion.usdoj.gov/GDP/(DEA-DC022)(DEA068)%20DEA%20SAMHSA%20buprenorphine%20telemedicine%20%20(Final)%20+Esign.pdf (Accessed March 31, 2020)

[10] Sarac NJ Sarac BA Schoenbrunner AR Janis JE Harrison RK Phieffer LS. A review of state guidelines for elective Orthopaedic procedures during the COVID-19 outbreak. J Bone Joint Surg Am. (2020) 102:942–5. doi: 10.2106/JBJS.20.00510, PMID: 32282419 PMC7197340

[11] Ziedan E Simon KI Wing C (2020). Effects of state COVID-19 closure policy on NON-COVID-19 health care utilization. National Bureau of Economic Research. (Accessed July 30, 2020).

[12] Ali MM Teich JL Mutter R. The role of perceived need and health insurance in substance use treatment: implications for the affordable care act. J Subst Abus Treat. (2015) 54:14–20. doi: 10.1016/j.jsat.2015.02.002, PMID: 25753655

[13] Compton WM Gfroerer J Conway KP Finger MS. Unemployment and substance outcomes in the United States 2002-2010. Drug Alcohol Depend. (2014) 142:350–3. doi: 10.1016/j.drugalcdep.2014.06.012, PMID: 25042761 PMC4127107

[14] Webster F Rice K Bhattacharyya O Katz J Oosenbrug E Upshur R. The mismeasurement of complexity: provider narratives of patients with complex needs in primary care settings. Int J Equity Health. (2019) 18:107. doi: 10.1186/s12939-019-1010-6, PMID: 31272466 PMC6611020

[15] Bluthenthal RN Simpson K Ceasar RC Zhao J Wenger L Kral AH. Opioid withdrawal symptoms, frequency, and pain characteristics as correlates of health risk among people who inject drugs. Drug Alcohol Depend. (2020) 211:107932. doi: 10.1016/j.drugalcdep.2020.107932, PMID: 32199668 PMC7259345

[16] Public Health Emergency Determination that a public health emergency exists. (2020) Available at: https://www.phe.gov/emergency/news/healthactions/phe/Pages/2019-nCoV.aspx (Accessed January 31, 2020).

[17] Services UDoHaH Notification of enforcement discretion for telehealth remote communications during the COVID-19 Nationwide public health emergency. (2020) Available at: https://www.hhs.gov/hipaa/for-professionals/special-topics/emergency-preparedness/notification-enforcement-discretion-telehealth/index.html

[18] Substance Abuse and Mental Health Services Administration Considerations for the care and treatment of mental and substance use disorders in the COVID-19 epidemic. (2020) Available at: https://www.samhsa.gov/sites/default/files/considerations-care-treatment-mental-substance-use-disorders-covid19.pdf (Accessed March 20, 2020).

[19] Leppla IE Gross MS. Optimizing medication treatment of opioid use disorder during COVID-19 (SARS-CoV-2). J Addict Med. (2020) 14:e1–3. doi: 10.1097/ADM.0000000000000678, PMID: 32412931 PMC7273937

[20] Shanthanna H Strand NH Provenzano DA Lobo CA Eldabe S Bhatia A. Caring for patients with pain during the COVID-19 pandemic: consensus recommendations from an international expert panel. Anaesthesia. (2020) 75:935–44. doi: 10.1111/anae.15076 32259288 PMC7262200

[21] Eccleston C Blyth FM Dear BF Fisher EA Keefe FJ Lynch ME. Managing patients with chronic pain during the COVID-19 outbreak: considerations for the rapid introduction of remotely supported (eHealth) pain management services. Pain. (2020) 161:889–93. doi: 10.1097/j.pain.0000000000001885, PMID: 32251203 PMC7172975

[22] Deer TR Sayed D Pope JE Chakravarthy KV Petersen E Moeschler SM. Emergence from the COVID-19 pandemic and the Care of Chronic Pain: guidance for the Interventionalist. Anesth Analg. (2020) 131:387–94. doi: 10.1213/ANE.0000000000005000, PMID: 32452905 PMC7258839

[23] Cohen SP Baber ZB Buvanendran A McLean BC Chen Y Hooten WM. Pain management best practices from multispecialty organizations during the COVID-19 pandemic and public health crises. Pain Med. (2020) 21:1331–46. doi: 10.1093/pm/pnaa127 32259247 PMC7184417

[24] Centers for Medicare & Medicaid Services (2020) Opening up America again: re-opening facilities to provide non-emergent non-COVID-19 healthcare: phase I. Available at: https://www.cms.gov/files/document/covid-flexibility-reopen-essential-non-covid-services.pdf

[25] Governor Newsom Releases Updated Industry Guidance. Office of Governor Gavin Newsom. (2020). Available at: https://www.gov.ca.gov/2020/05/07/governor-newsom-releases-updated-industry-guidance/

[26] Currie JM Schnell MK Schwandt H Zhang J. Prescribing of opioid analgesics and buprenorphine for opioid use disorder during the COVID-19 pandemic. JAMA Netw Open. (2021) 4:e216147. doi: 10.1001/jamanetworkopen.2021.6147, PMID: 33856474 PMC8050741

[27] Downs CG Varisco TJ Bapat SS Shen C Thornton JD. Impact of COVID-19 related policy changes on filling of opioid and benzodiazepine medications. Res Soc Adm Pharm. (2021) 17:2005–8. doi: 10.1016/j.sapharm.2020.06.003, PMID: 33317769 PMC7738763

[28] O'Donoghue AL Biswas N Dechen T Anderson TS Talmor N Punnamaraju A. Trends in filled naloxone prescriptions before and during the COVID-19 pandemic in the United States. JAMA Health Forum. (2021) 2:e210393. doi: 10.1001/jamahealthforum.2021.0393, PMID: 35977309 PMC8796899

[29] Demyttenaere K Bruffaerts R Lee S Posada-Villa J Kovess V Angermeyer MC. Mental disorders among persons with chronic back or neck pain: results from the world mental health surveys. Pain. (2007) 129:332–42. doi: 10.1016/j.pain.2007.01.022 17350169

[30] Jones CM McCance-Katz EF. Co-occurring substance use and mental disorders among adults with opioid use disorder. Drug Alcohol Depend. (2019) 197:78–82. doi: 10.1016/j.drugalcdep.2018.12.030 30784952

[31] Lacasse A Page MG Dassieu L Sourial N Janelle-Montcalm A Dorais M. Impact of the COVID-19 pandemic on the pharmacological, physical, and psychological treatments of pain: findings from the chronic pain & COVID-19 Pan-Canadian study. Pain Rep. (2021) 6:e891. doi: 10.1097/PR9.0000000000000891, PMID: 33598594 PMC7880148

[32] Nieto R Pardo R Sora B Feliu-Soler A Luciano JV. Impact of COVID-19 lockdown measures on Spanish people with chronic pain: an online study survey. J Clin Med. (2020) 9:215–29. doi: 10.3390/jcm9113558, PMID: 33167322 PMC7694344

[33] Wosik J Fudim M Cameron B Gellad ZF Cho A Phinney D. Telehealth transformation: COVID-19 and the rise of virtual care. J Am Med Inform Assoc. (2020) 27:957–62. doi: 10.1093/jamia/ocaa067, PMID: 32311034 PMC7188147

[34] Robinson J Borgo L Fennell K. The Covid-19 pandemic accelerates the transition to virtual care. NEJM Catalyst. (2020) 1:1–11. doi: 10.1056/CAT.20.0399

[35] Jones CM Diallo MM Vythilingam M Schier JG Eisenstat M Compton WM. Characteristics and correlates of U.S. clinicians prescribing buprenorphine for opioid use disorder treatment using expanded authorities during the COVID-19 pandemic. Drug Alcohol Depend. (2021) 225:108783. doi: 10.1016/j.drugalcdep.2021.108783, PMID: 34049102 PMC9186054

